# Diagnostic Challenges in Hemophagocytic Lymphohistiocytosis, a Rare, Potentially Fatal Disease: Two Case Studies

**DOI:** 10.3390/jcm13061643

**Published:** 2024-03-13

**Authors:** Marcela Daniela Ionescu, Bianca Prajescu, Roxana Taras, Nicoleta Popescu, Ruxandra Vidlescu, Mihaela Smarandoiu, Loredana-Elena Rosca, Augustina Enculescu, Elena Camelia Berghea, Claudia Lucia Toma

**Affiliations:** 1 Department of Pediatrics, Carol Davila University of Medicine and Pharmacy, 020021 Bucharest, Romania; daniela.ionescu@umfcd.ro (M.D.I.); roxana.taras@drd.umfcd.ro (R.T.); camelia.berghea@umfcd.ro (E.C.B.); 2 Marie Curie Emergency Children’s Hospital, 041451 Bucharest, Romania; ped_nicoleta.popescu@yahoo.com (N.P.); ruxandra.vidlescu@gmail.com (R.V.); mihaelagurgu88@gmail.com (M.S.); loredanatufaru@yahoo.com (L.-E.R.); augustina.enculescu@gmail.com (A.E.); 3 Department of Pneumology, Carol Davila University of Medicine and Pharmacy, 020021 Bucharest, Romania; claudiatoma@yahoo.co.uk; 4 Marius Nasta Institute of Pneumology, 010024 Bucharest, Romania

**Keywords:** hemophagocytic lymphohistiocytosis, ferritin, sepsis

## Abstract

Hemophagocytic lymphohistiocytosis (HLH) is a rare, and potentially fatal, syndrome, characterized by immune system dysregulation, with excessive activation of the macrophages and cytotoxic T cells. It can be classified into primary (genetic) and secondary (acquired) forms. HLH presents with fever, hepatosplenomegaly, cytopenia, and hyperferritinemia, with involvement of various organs. The initial symptoms of HLH are non-specific, but as, if untreated, it can progress rapidly to multiorgan failure, timely diagnosis is essential. We present here two cases of HLH in infants that illustrate the importance of early diagnosis and appropriate treatment, along with a short review of HLH.

## 1. Introduction

Hemophagocytic lymphohistiocytosis (HLH) or hemophagocytic syndrome is a rare, life-threatening condition caused by abnormal immune system activation. HLH is characterized clinically by high fever and hepatosplenomegaly. The laboratory findings are pancytopenia, hyperferritinemia, hypertriglyceridemia, hypofibrinogenemia, and increased macrophage activation with hemophagocytosis [1]. The intensive macrophage and lymphocyte activation leads to the release of pro-inflammatory cytokines, including tumor necrosis factor-α (TNF-α), interferon-gamma (IFN-γ), interleukin (IL)-6, IL-8, IL-12, IL-18, and macrophage colony-stimulating factor (M-CSF) [2]. Because of its clinical similarity to other inflammatory conditions and the non-specific initial symptoms, the timely diagnosis of HLH can be challenging, but is of vital importance [3,4]. 

## 2. Literature Review

### 2.1. Epidemiology and Incidence Trends in HLH

HLH is considered to be primarily a pediatric disease, with the highest incidence in infants aged < 3 months, but it can affect individuals at any age. No gender difference in incidence has been observed [5], but an increased susceptibility has been noted among Japanese patients [6,7]. 

HLH can manifest as either a familial or a secondary disorder and may be initiated by a variety of events that disrupt the immune homeostasis, including infective agents, most frequently the Epstein–Barr virus (EBV), or may be related to immunosuppressive conditions (e.g., malignancy, autoimmune disease) [8].

Familial HLH (FHL) is an autosomal recessive condition with a maximum incidence during infancy. The genetic basis consists of abnormalities in the function of cytotoxic T lymphocytes (CTLs) and natural killer (NK) cells. The mutations manifest predominantly in the perforin gene or genes crucial for the functioning of CTLs. The trigger can be an acute infection, but sometimes an infectious agent may not be evident [9,10,11]. 

Secondary HLH can manifest at any age and is distinguished by acquired deficiencies in the cytotoxic function of the T-lymphocytes and NK cells. It is linked to an episode of immune activation, such as an infection, or an episode of immune suppression, as observed in malignancies, rheumatic diseases and other autoimmune disorders [12]. The infective agents that are known to trigger secondary HLH are viruses, most often EBV, but also cytomegalovirus (CMV) and human immunodeficiency virus (HIV), bacteria (e.g., tick-borne bacteria, mycoplasma), fungi (e.g., histoplasma), and parasites (e.g., Leishmania) [13]. The rheumatic diseases associated with HLH are systemic juvenile idiopathic arthritis (SJIA), systemic lupus erythematosus (SLE), dermatomyositis, and Kawasaki disease [4]. Secondary HLH presenting in the context of rheumatic disease is named the macrophage activation syndrome (MAS) [14]. Malignancies, most frequently lymphoma, can also trigger secondary HLH [15]. Patients may exhibit the clinical syndrome of HLH in conjunction with an undiagnosed underlying malignancy (new onset), or they may experience HLH during the treatment of a known malignancy (on therapy), typically in the context of an infection [4].

### 2.2. The Genetic Background of HLH

HLH is often associated with genetic irregularities that affect lymphocyte cytotoxicity. It was widely believed, initially, that the symptoms of genetically induced HLH presented in infancy and early childhood. With the increased availability of genetic testing, however, it has become apparent that the inaugural significant episode of HLH can occur at any stage of life, ranging from infancy to as late as the seventh decade [16]. Genetic data can be of value in assessing the probability of reoccurrence, the need for hematopoietic cell transplantation (HCT), and the risk of HLH among family members.

Genetic HLH can be FHL, which is an autosomal recessive disorder, or it may be associated with a genetic immunodeficiency syndrome [17]. Concerning FHL, mutations have been discovered in a group of genes that play a role in the exocytosis of cytoplasmic granules, resulting in the perforin-mediated destruction of target cells, such as perforin encoding gene 1 (PRF1), mammalian uncoordinated13 (MUNC13-4), syntaxin 11 (STX11) and syntaxin-binding protein 2 (STXBP2) [18]. Based on these genetic defects, FHL can be subdivided into four types, from FHL-2 to FHL-5. For FHL-1, the gene has not yet been identified [19]. The most frequent mutation identified is PRF1, which is found in almost 30% of cases of FHL and is specific for FHL-2 [20]. 

Other genetic defects incriminated in HLH are those that are associated with immunodeficiency syndromes, in which situation, the patients are unable to fight off an invasive pathogen, resulting in FHL manifestations [21]. Such genes are SH2DIA, associated with X-linked lymphoproliferative syndrome (XLP) 1; XIAP, associated with XLP2; RAB27A, associated with Griscelly syndrome type 2; LYST, associated with Chediak–Higashi syndrome; and AP3BI, associated with Hermansky-Pudlak syndrome [22].

### 2.3. Immunological Mechanisms in HLH

HLH is characterized by multisystem inflammation that occurs when regulatory pathways responsible for down-regulation of the immune response experience disruption or are overwhelmed. This leads to prolonged and excessive activation of antigen-presenting cells (macrophages, histiocytes) and T lymphocytes [23].

Macrophages, originating from circulating monocytes, serve as antigen-presenting cells by presenting foreign antigens to lymphocytes [24]. The role of NK cells and CTLs is to eradicate compromised, stressed or infected host cells, including macrophages, often in response to viral infection or malignancy [25]. 

The NK cells and CTLs usually eliminate the targeted cells through perforin-dependent cytotoxicity. The majority of the genetic abnormalities in cases of familial HLH are related to this process [26]. 

In HLH, the NK cells and CTLs are unable to eliminate the activated macrophages, which release large amounts of cytokines that are responsible for multiorgan failure [27]. The cytokines involved are TNF-α, IFN-γ, chemokine ligand 9 (CXCL9), IL-6, IL-10, IL-12, and IL-18 [20]. IFN-γ and CXCL9 are used as biomarkers of the activity of the disease, and can be therapeutic targets in the treatment of HLH [28]. Recently, it was reported that a value of IL 18 > 24,000 pg/mL could distinguish MAS from FHL or other hyperferritinemic syndromes [29]. 

In addition to the release of cytokines, macrophages can phagocytize blood cells, a process named hemophagocytosis. Hemophagocytosis, which can be detected on tissue biopsy from the spleen, liver or lymph nodes, or bone marrow aspiration, is a hallmark of activated macrophages, but it is not pathognomonic for the diagnosis of HLH [30].

### 2.4. Diagnosis

Typically, patients with HLH present with prolonged fever and hepatosplenomegaly. In 30–73% of cases, patients also have neurological symptoms, such as seizures, meningismus, ataxia, and a change in mental status. The involvement of the central nervous system (CNS) is associated with a poorer outcome [31]. Patients may exhibit diverse skin manifestations, such as widespread maculopapular erythematous rash, generalized erythroderma, edema, panniculitis, morbilliform erythema, petechiae, and purpura. Features suggestive of Kawasaki disease may be displayed, characterized by an erythematous rash, conjunctivitis, redness of the lips, and enlarged cervical lymph nodes [32]. In the most severe cases of HLH, multiple organ dysfunction may result from the cytokine storm, with acute respiratory distress syndrome (ARSD), renal failure, severe hypotension, and hemorrhages [6]. 

Blood tests may reveal cytopenia (i.e., anemia, thrombocytopenia), hyperferritinemia, liver dysfunction, hypofibrinogenemia, and hypertriglyceridemia [33]. Hyperferritinemia can also be associated with iron overload or other inflammatory diseases. For the diagnosis of HLH, a value of >500 μg/L was associated with >90% sensitivity, but values of >2000 μg/L have a robust specificity [34]. Most patients develop liver dysfunction, manifested by raised blood levels of bilirubin, the liver enzymes alanine transaminase (ALT), aspartate aminotransferase (AST) and gamma-glutamyl transferase (GGT), and lactate dehydrogenase (LDH). In severe liver dysfunction, hypertriglyceridemia and coagulation abnormalities may result from impaired synthetic function or disseminated intravascular coagulation [35]. Additional findings suggestive of HLH are raised levels of C-reactive protein (CRP) and D-dimer, and hyponatremia and hypoalbuminemia [36].

The immunological response can be evaluated by immunoglobulin levels, which can be variable, and investigation of the lymphocyte subsets, which may show decreased numbers of B cells or NK cells. More specific tests are the dosage of soluble IL-2 receptor and tests of NK function, which are available only in specialized centers. In HLH, the soluble IL-2 receptor (IL-2R or CD25) level is high, and NK function is low or absent [25].

Hemophagocytosis may be detected on bone marrow aspiration or biopsy from the lymphoid organs, although in the early stages, the bone marrow may be negative [23]. 

In order to establish a timely diagnosis, the Histiocyte Society developed diagnostic guidelines, including clinical and paraclinical findings, in 1994, which were amended in 2004, as shown in Table 1 [37]. According to the updated diagnostic criteria outlined in the HLH-2004 protocol, the assumption of HLH is warranted if there is either (A) a genetic diagnosis consistent with HLH or (B) five of the eight diagnostic criteria are met [34].

The HLH-2004 criteria may be useful in guiding the diagnosis, but there are certain limitations. In the initial stages of the disease, some patients present with symptoms and signs that do not meet five of the eight criteria, and some may never meet these criteria, especially those with atypical manifestations, such as CNS disease or acute liver failure [38]. Estimation of soluble CD25 or the evaluation of NK function may not be available in routine practice [39]. In addition, the HLH-2004 criteria cannot differentiate between the primary and the secondary forms of HLH [40]. 

For the diagnosis of secondary HLH, Fardet and colleagues proposed an algorithm, as shown in Table 2, that they named HScore. The HScore includes a history of immunosuppression, clinical features (raised temperature and organomegaly), and laboratory features (cytopenia, ferritin, triglyceride, fibrinogen, aspartate aminotransferase, and hemophagocytosis on bone marrow aspirate). The probability of HLH according to HScore is shown in Table 3 [39]. The HScore is mostly used in the diagnosis of adults with secondary HLH, but several recent studies showed that the HScore might be a better predictor than the HLH-2004 criteria in the diagnosis of secondary HLH in children [41]. 

In the case of rheumatic diseases such as SJIA, the diagnosis of MAS can be established using the 2016 classification criteria proposed by Ravelli and colleagues. The 2016 classification criteria refers to a patient suspected or diagnosed with SJIA, febrile, with a ferritin value > 684 ng/mL associated with at least two of the following: thrombocytopenia < 181,000/mm^3^, AST > 48 U/L, triglycerides > 156 mg/dL, and fibrinogen < 360 mg/dL (see Table 4) [42].

### 2.5. Advances in the Treatment of HLH

Without treatment, the survival of patients with HLH is approximately 2 months [5]. Given the fatal nature of HLH, specific treatment must be initiated promptly when the clinical suspicion is high, even if not all of the criteria are met. The treatment consists of chemotherapy and immunosuppressive drugs, specifically etoposide (VP-16), steroids, and antithymocyte globulin (ATG). The only curative method, especially in the case of FHL, is HCT [23]. 

In 1994, the Histiocyte Society created the first protocol for HLH treatment, the HLH-94 protocol, which led to a survival rate of 55%. This protocol consisted of an 8-week induction treatment with VP-16, dexamethasone, and intrathecal methotrexate (MTX) or hydrocortisone (HC) for those with CNS involvement. After induction treatment, continuation treatment, consisting of dexamethasone, VP-16 and cyclosporine A, was recommended up until HCT could be given, especially for those with familial, severe or relapsing forms of HLH [32,33]. 

**Figure 1:**
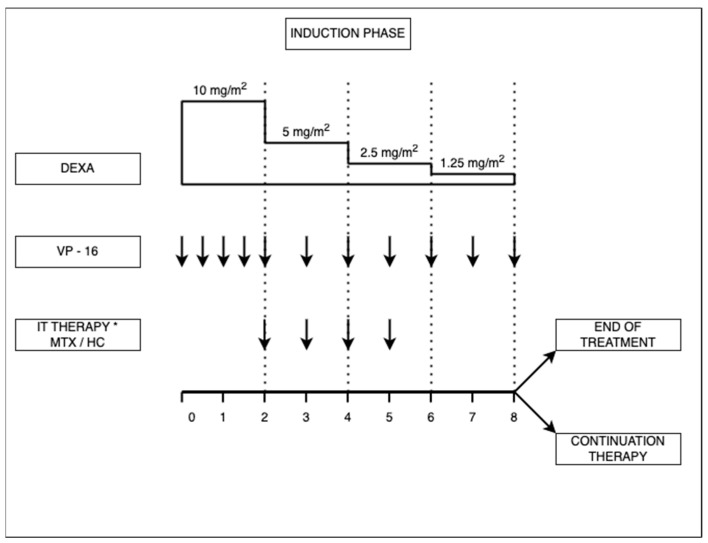
HLH-94 treatment scheme for hemophagocytic lymphohistiocytosis (adapted from Jordan et al. [32]). DEXA: dexamethasone, at an initial dose of 10 mg/m^2^, daily, followed by halving of the dosage every two weeks; VP-16:etoposide, administered twice weekly in the first two weeks, and once a week thereafter, at a dose of 150 mg/m^2^ or 5 mg/kg for children weighing < 10 kg; IT: intrathecal treatment, * in the case of CNS involvement, with –methotrexate (MTX) or –hydrocortisone (HC), in dosages of: <1 year—6 mg MTX and 8 mg HC, 1–2 years—8 mg MTX and 10 mg HC, 2–3 years—10 mg MTX and 12 mg HC, >3 years—12 mg MTX and 15 mg HC.

In the induction phase, dexamethasone may be given orally or intravenously, starting with an initial dose of 10 mg/m^2^, followed by halving of the dosage every two weeks. VP-16 is administered twice weekly in the first two weeks, then once a week, at a dose of 150 mg/m^2^ or 5 mg/kg for children weighing < 10 kg. The VP-16 dose must be adjusted for patients with renal failure or hepatic dysfunction. If there is no remaining liver function, alemtuzumab could be an alternative for VP-16. For patients with CNS involvement, intrathecal therapy with MTX or HC must be started as soon as lumbar puncture can be performed safely, then administered weekly. The dosage is related to the patient’s age as follows: <1 year—6 mg MTX and 8 mg HC, 1–2 years—8 mg MTX and 10 mg HC, 2–3 years—10 mg MTX and 12 mg HC, and >3 years—12 mg MTX and 15 mg HC (see Figure 1) [43]. 

The continuation treatment of the HLH-94 protocol consists of dexamethasone 10 mg/m^2^/day for 3 days, every second week, VP-16 150 mg/m^2^ every second week, and cyclosporine at a daily dosage of 6 mg/kg, administered in divided doses, aiming for a target trough level of 200 mcg/L [43].

**Figure 2:**
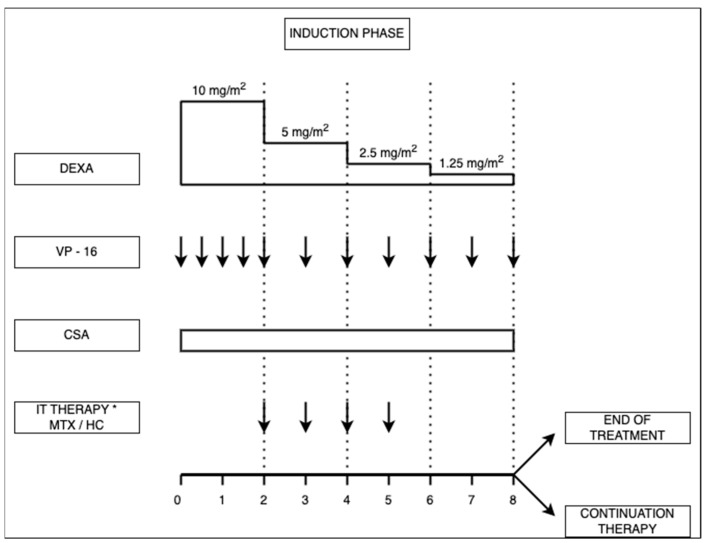
HLH-2004 treatment scheme for hemophagocytic lymphohistiocytosis (adapted from Sun Young Park et al. [47]). DEXA: dexamethasone at an initial dose of 10 mg/m^2^, daily, followed by halving of the dosage every two weeks; VP-16: etoposide, administered twice weekly in the first two weeks, and once a week thereafter, at a dose of 150 mg/m^2^ or 5 mg/kg for children weighing < 10 kg; CSA: cyclosporine A at a dose of 6 mg/kg/day; IT: intrathecal therapy * if CNS involved with MTX: methotrexate or HC: hydrocortisone must be started, <1 year—6 mg MTX and 8 mg HC, 1–2 years—8 mg MTX and 10 mg HC, 2–3 years—10 mg MTX and 12 mg HC, >3 years—12 mg MTX and 15 mg HC.

Over time, with new trials, modifications have been made. The HLH-2004 protocol, for example, proposes more intensive treatment, moving the cyclosporin to the beginning of the induction, with the aim of reducing mortality and morbidity pre-HCT. In HLH-2004 the induction treatment consists of dexamethasone, VP-16, and intrathecal treatment with MTX/HC at the same dosage as for HLH-94, adding cyclosporine A at a dose of 6 mg/kg/day. The continuation treatment is similar to that of the HLH-94 scheme, but with a total treatment period of 40 weeks instead of 52 weeks (see Figure 2) [33]. In addition, one single-center study proposes to treat patients with HLH with corticosteroids and ATG, followed rapidly by HCT for those with FHL, but the outcome has not yet been studied [44].

In case of refractory response, salvage therapy can be administered consisting of adriamycin, cyclophosphamide, vincristine, procarbazine and prednisolone (ACOPP), or adriamycin, bleomycin, vinblastine and prednisolone (ABVD) [45].

In patients with MAS, the treatment consists of corticosteroids combined with cyclosporine, and refractory cases might be treated with a VP-16 regimen. In many cases, intravenous immunoglobulins (IVIG) can be used, but only in combination with other molecules [46].

## 3. Case Presentations

### 3.1. Case 1

A 2-month-old male infant with pancytopenia was referred from a regional hospital for further evaluation. He had a history of fever and diarrhea for a week, treated with antibiotics with no improvement.

He was the second child of non-consanguineous parents, with no significant perinatal history (normal pregnancy, vaginal delivery at gestational age 39 weeks, birth weight 2700 g, Apgar Score 9, good neonatal development). He was breast fed with good food tolerance and satisfactory weight gain, and had no history of other acute illnesses.

On physical examination, he presented good tone and reactivity. He had fever, and cutaneous and mucous pallor, but no skin rash. Respiratory and cardiovascular system examination was normal. His abdomen was enlarged by splenomegaly (inferior pole of spleen palpable at the superior iliac crest) and hepatomegaly (liver edge palpable 2 cm below the right costal rim). Upon observation, he had good digestive oral tolerance, but diarrhea (>7 stool emissions/24 h). 

The laboratory examination, as shown in Table 5, revealed pancytopenia, with severe anemia: Hemoglobin (Hb) 5.6 g/dL, leukopenia: white blood cells (WBC) 3640/μL, thrombocytopenia: platelets 16,000/μL), hyperferritinemia: 1, ferritin 342 μg/L, hypertriglyceridemia 360 mg/dL, and hypofibrinogenemia 77 mg/dL. The liver enzymes, urea and creatinine were within normal range. The inflammatory markers were slightly increased: CRP 9.75 mg/L, procalcitonin 0.256 ng/mL and IL-6 16 pg/mL. The peripheral blood smear showed erythrocyte poikilocytosis, red blood cells with punctate basophilia, polychromatophilic macrocytes, no abnormal leukocyte morphology, and marked platelet anisocytosis.

Viral serology was negative for coronavirus, CMV, EBV, parvovirus B19, HIV, rubella, measles, adenovirus, and rotavirus. Blood culture, stool culture, and pharyngeal exudate culture were also negative (see Table 6).

The bone marrow aspirate showed moderately increased cellularity, with a predominance of the erythrocyte series, approximately 68%, with normal maturation: proerythroblasts 1%, basophilic erythroblasts 6%, polychromatophilic erythroblasts 37%, and oxyphilic erythroblasts 24%. The granulocyte series was approximately 30%, with normal maturation. Very rare lymphocytes, rare monocytes, and no atypical cells were observed. The platelet series was represented by very rare thrombocytogenic megakaryocytes.

The chest X-ray showed no lesions. The abdominal ultrasound confirmed splenomegaly with a longitudinal diameter of 10 cm and an accessory spleen of 8 mm. Cardiac echocardiography revealed a small atrial septum defect with no hemodynamic impact. 

The initial diagnosis was digestive sepsis and the patient was treated with broad-spectrum antibiotics (ceftazidime 150 mg/kg/day for 12 days, teicoplanin 16 mg/kg/day as the loading dose, then 8 mg/kg/day for 17 days, meropenem 60 mg/kg/day for 7 days) and antimycotic therapy (fluconazole 6 mg/kg/day as the loading dose, then 3 mg/kg/day for 16 days). Because of the persistent fever, splenomegaly, and pancytopenia, treatment with systemic dexamethasone (0.6 mg/kg/day for 9 days then progressively decreasing doses for 9 days) was also initiated, along with intravenous human immunoglobulin products (0.5 mg/kg/day, 2 administrations). The anemia, thrombocytopenia, and hypofibrinogenemia persisted, and transfusion of blood products became necessary. 

Despite the intensive treatment, the symptoms and the laboratory abnormalities persisted. The differential diagnosis excluded viral infections (negative serology), bacterial infections (blood culture, pharyngeal exudate, stool culture negative), tuberculosis (protein purified derivate skin test negative, chest X-ray without lesions), malignant hemopathies (peripheral blood smear and bone marrow aspiration without atypical cells), and metabolic diseases (no specific cells on blood smear analysis).

In view of the clinical and paraclinical data, with persistent fever, splenomegaly, pancytopenia, hypofibrinogenemia, a high serum triglyceride level, and a high serum ferritin level, we considered the possibility of FHL, as five of the eight diagnostic criteria were present. This diagnosis was later genetically confirmed, as a homozygous UNC13D mutation was detected.

Chemotherapy was therefore initiated, according to the HLH 2004 protocol (see Figure 2), following which a slowly favorable response was observed. Because the homozygous UNC13D mutation was found, the only curative treatment was HCT, and the patient was transferred to another clinic for medullar allogenic transplantation. After transplantation, the evolution was favorable. The fever and alimentary tract symptoms disappeared, and the blood count and blood levels of ferritin and triglycerides returned to within the normal range, but the hepatosplenomegaly persisted. 

### 3.2. Case 2

An infant girl aged 15 months presented with a 10-day history of high fever and cough. Because of persistent fever under antipyretic treatment, she had received a 7-day course of antibiotics prescribed by her general practitioner, despite which her temperature remained above 39 °C, and she became apathetic and anorexic and developed a paroxysmal cough. In the 24 h before admission, she showed marked respiratory distress and deterioration of her general condition. Previously, she had been a healthy child, with no relevant medical history or family history.

Upon admission, she was afebrile and conscious, with periods of drowsiness and agitation. She was pale, with a few petechiae on the face and the anterior thorax. 

She presented groaning, paroxysmal coughing, dyspnea, and tachypnea, with a respiratory rate (RR) of 50 breaths/min, oxygen saturation (SpO_2_) of 88% in room air and 92–94% with 4 L/min O_2_, a heart rate (HR) of 100 beats/min, and a blood pressure (BP) of 100/65 mmHg. Auscultation revealed an absence of sounds over the right hemithorax and fine crackles in the left upper lobe. Abdominal examination revealed enlargement of the liver and spleen.

Laboratory tests showed pancytopenia (leukopenia with lymphopenia, neutropenia, anemia, and thrombocytopenia), marked indications of inflammatory syndrome (CRP 247 mg/L, procalcitonin 10 ng/mL) and metabolic acidosis (pH 7.32, bicarbonate 16.3 mmol/L), and hyponatremia (127.5 mmol/L). Signs of a coagulation disorder were found, namely prolonged prothrombin time (PT), activated partial thromboplastin time (APTT), and international normalized ratio (INR) (Table 7). Blood cultures were negative.

**Figure 3:**
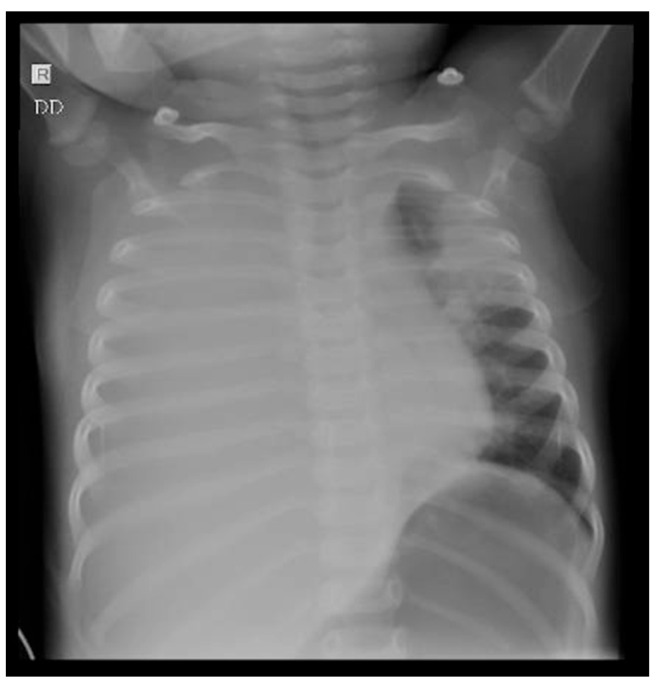
A 15-month-old female infant with hemophagocytic lymphohistiocytosis: pulmonary X-ray showing massive opacity of the right hemithorax, consolidation of the left upper lobe, left perihilar infiltrate.

**Figure 4:**
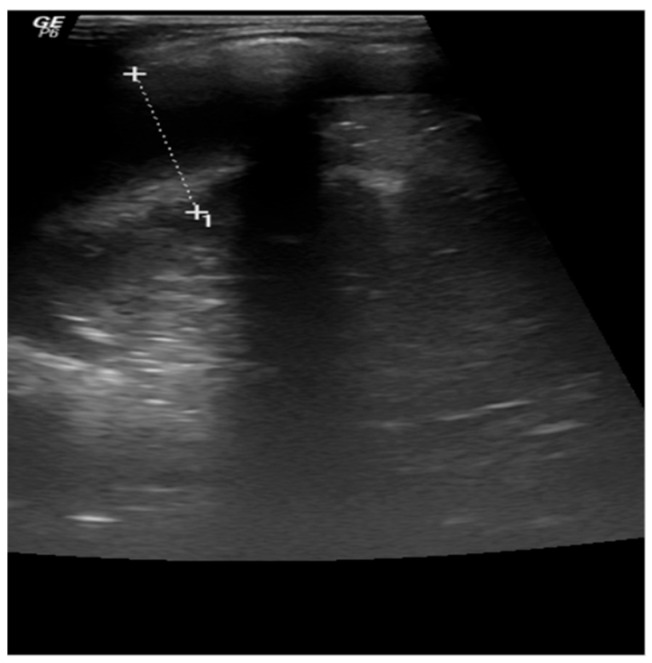
A 15-month-old female infant with hemophagocytic lymphohistiocytosis: thoracic ultrasound showing right-sided pleurisy in medium quantity—2.5 cm.

Chest X-ray revealed massive opacity of the right hemithorax, left upper lobe consolidation, and left perihilar and basal infiltrate (Figure 3). Chest ultrasound showed pleural effusion of the right hemithorax of 2.5 cm, right upper and lower lobe consolidation with air bronchogram (Figure 4).

All the laboratory and imaging data suggested the diagnosis of respiratory sepsis from an unknown pathogen, with multilobar pneumonia complicated with large pleural effusion.

Upon admission, emergency treatment was administered, consisting of oxygen therapy, fluid resuscitation, broad-spectrum antibiotics (Meropenem 120 mg/kg/day, Vancomycin 50 mg/kg/day), steroids (Methylprednisolone 2 mg/kg/day), diuretics (Furosemide 1 mg/kg/dose), platelets transfusion (5 mL/kg), and vitamin K (10 mg).

The course was unfavorable with rapid deterioration, marked respiratory effort, absence of sounds over the right hemithorax and diminished sounds over the left hemithorax, SpO_2_ 86% with oxygen therapy 4 L/min, RR 55 breaths/min, central and peripheral cyanosis, capillary refill time > 3 s, tachycardia, HR 180 beats/min, BP 90/50 mmHg, “coffee grounds” nasogastric fluid, and episodes of marked psychomotor agitation or drowsiness.

As shown in Table 7, repeat laboratory tests showed decreasing values of Hb and persistence of lymphopenia and thrombocytopenia. The ESR was normal, but the CRP level was raised, and the ferritin recorded a notably high value of 4596 μg/L. Fibrinogen was within a normal range. The other tests revealed the evolution towards multiple organ failure, including hepatic cytolysis, nitrogen retention syndrome, and myocardial damage. The immunogram showed a slightly low level of IgG.

**Figure 5:**
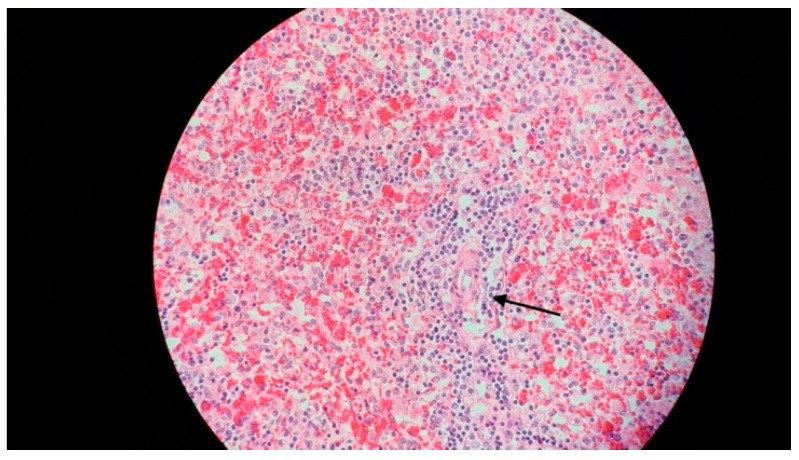
A 15-month-old female infant with hemophagocytic lymphohistiocytosis: spleen biopsy (hematoxylin and eosin, 400×), showing signs of splenic sinusoidal hemophagocytosis (arrow).

**Figure 6:**
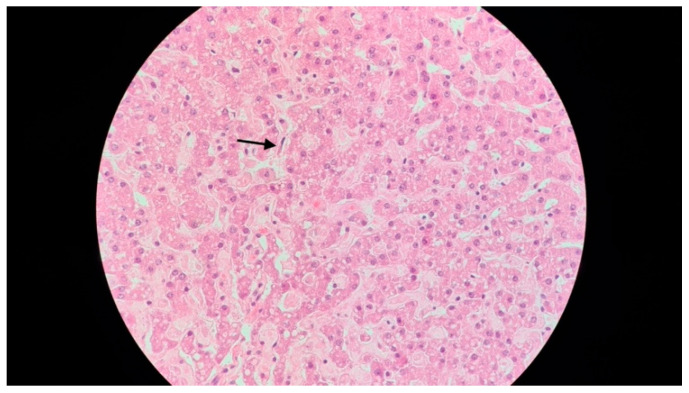
A 15-month-old female infant with hemophagocytic lymphohistiocytosis: liver biopsy (hematoxylin and eosin, 400×), showing signs of sinusoidal hemophagocytosis (arrow).

In spite of the intensive treatment, 16 h after admission, the patient presented cardiorespiratory arrest, which did not respond to resuscitation maneuvers. In view of the rapid evolution towards death, the initial symptoms and clinical findings (fever, hepatosplenomegaly), and the laboratory findings, HLH was suspected, and the bone marrow aspiration performed postmortem, during the autopsy, revealed very frequent macrophages with hemophagocytic elements suggestive of HLH, as did biopsy specimens of the spleen (Figure 5) and the liver (Figure 6).

The necropsy established the diagnoses of right-sided pneumonia with right serofibrinous pleurisy and pulmonary hemorrhage, dilated cardiomyopathy, hepatosplenomegaly, hemorrhagic gastritis, and stasis nephropathy.

## 4. Discussion

In the first case presented above, the diagnosis was difficult. The clinical and paraclinical data suggested the diagnosis of digestive sepsis. Initially, broad-spectrum antibiotics were administered, followed by steroids and immunoglobulins, but the symptoms worsened, so further assessment was performed to identify possible causes, such as viral infection, bacterial infection, fungal infection, or malignancy. 

The exclusion of these etiologies, and the persistent symptomatology oriented the diagnosis to HLH, for which five of the eight criteria were met (persistent fever, splenomegaly, pancytopenia, hypofibrinogenemia, hypertriglyceridemia, and hyperferritinemia), but without evident hemophagocytosis on the bone marrow aspirate. As other authors affirmed, hemophagocytosis may not be observed in the bone marrow aspirate in the initial stages of the disease [23,48]. The presence of hemophagocytosis would have established the diagnosis sooner, but its absence in this case is an important reminder to consider HLH in a severe clinical situation even with a biopsy negative for hemophagocytosis.

Given the age of the infant in the first case (two months), it appeared more likely to be a familial form of HLH, and indeed, genetic testing revealed the presence of the UNCD13D mutation. Mutation of the UNCD13D gene is responsible for abnormal MUNC13-4 protein synthesis, a protein involved in the perforin-mediated cytolysis. This mutation classifies this subtype of HLH as FHL-3 [49], which constitutes 10–32% of cases of genetically based HLH [50]. It has been observed that in FHL-3, the CNS is more frequently affected than in the other forms [51], although in the case presented here, there was no evidence of CNS involvement. 

To achieve remission, immunosuppressive treatment was initiated according to the HLH 2004 protocol, comprising dexamethasone, cyclosporine A, and etoposide. In the absence of neurological symptoms, intrathecal methotrexate was not needed in this case [52]. Genetically based HLH cases tend to relapse in spite of immunosuppressive treatment. The only curative treatment is HCT, which has a high mortality risk, due to myeloablative conditioning [53]. The HCT was performed on our patient in another clinic with satisfactory results. 

In the second case described here, the diagnosis was more challenging, because the symptoms were non-specific, and the evolution was rapidly fatal. Upon initial analysis of the clinical presentation (fever, cough, dyspnea, and hepatosplenomegaly), the initial laboratory findings (pancytopenia, markedly raised inflammatory markers, and coagulation disorder), and the imaging findings (pleural effusion, consolidation of left upper lobe, right upper and lower lobe), the most likely diagnosis was respiratory sepsis complicated by multiorgan failure. 

Because of the rapid deterioration of the patient, extensive laboratory tests were performed, some of which could have oriented the diagnosis towards HLH. For example, the fibrinogen level and the ESR were within the normal range, in contrast to the markedly elevated values of CRP and procalcitonin. Fibrinogen, like CRP and procalcitonin, is an acute-phase reactant, and is usually elevated in inflammatory diseases. In contrast, in HLH fibrinogen can be decreased, as can the ESR, which is low secondary to the decrease in fibrinogen [36]. 

Ferritin was greatly increased (4596 μg/L). A raised ferritin level is common in patients with HLH, but it is nonspecific. A value of ferritin > 500 μg/L is one of the criteria of HLH-2004, but at the moment it was found in our patient, she fulfilled only four of the eight criteria (fever, splenomegaly, pancytopenia, and hyperferritinemia). In our center, CD25 and NK cell activity cannot be analyzed. In addition, because of the coagulation disorders and the risk of hemorrhage, pleural puncture and bone marrow biopsy could not be performed. 

Unfortunately, the patient’s condition deteriorated rapidly leading to death within 24 h of admission. Bone marrow aspiration was performed during autopsy and revealed the presence of hemophagocytosis. Hemophagocytosis is a hallmark of HLH, but it can be also found in critically ill patients with infections or autoimmune diseases [54]. 

In the second case presented here, the diagnosis of HLH was established postmortem. For this patient, the HLH appeared to be secondary to a respiratory infection, but considering the age of the patient (15 months) it is difficult to distinguish whether it was an FHL triggered by infection or if it was a secondary form of HLH. Genetic testing would have helped in identifying genetic abnormalities.

## 5. Conclusions

HLH, though uncommon, is a severe condition that, without timely, appropriate treatment, can lead rapidly to death. Its resemblance to other serious illnesses may cause it to be overlooked. We present two cases of HLH in infants from our clinic to illustrate the complexities of diagnosing HLH and to underscore the critical significance of prompt commencement of appropriate treatment.

## Figures and Tables

**Table 1:** Diagnostic criteria for hemophagocytic lymphohistiocytosis, according to the Histiocyte Society HLH-2004 study.

A. **Genetic Defect Consistent with Hemophagocytic Lymphohistiocytosis (HLH), or** B. **5 of the 8 Following Criteria:**
Fever, daily
2. Splenomegaly
3. Bicytopenia (Hb < 9 g/dL, thrombocytes < 100 × 10^9^/L, neutrophils < 10^9^/L)
4. Hypertriglyceridemia (>265 mg/dL) and/or hypofibrinogenemia (<150 mg/dL)
5. Hyperferritinemia (>500 μg/L)
6. Soluble CD25 (IL-2R) above normal limits for age
7. Low or absent natural killer (NK) cell function
8. Hemophagocytosis in bone marrow, lymph nodes, spleen, or cerebrospinal fluid (CSF)

**Table 2:** The HScore algorithm for the diagnosis of secondary hemophagocytic lymphohistiocytosis (HLH) (adapted from Fardet et al. [39]).

Parameter	Points
History of immunosuppression *	No—0 points Yes—18 points
Temperature	<38.4 °C—0 points 38.4–39.4 °C—33 points >39.4 °C—49 points
Organomegaly	None—0 points Hepatomegaly or splenomegaly—23 points Hepatomegaly and splenomegaly—38 points
Cytopenia **	1 lineage—0 points 2 lineages—24 points 3 lineages—34 points
Ferritin	<2000 ng/mL—0 points 2000–6000 ng/mL—35 points >6000 ng/mL—50 points
Triglyceride	<132 mg/dL—0 points 132–350 mg/dL—44 points >350 mg/dL—64 points
Fibrinogen	>250 mg/dL—0 points ≤250 mg/dL—30 points
Alanine aminotransferase	<30 UI/L—0 points ≥30 UI/L—19 points
Hemophagocytosis on bone marrow aspirate	No—0 points Yes—35 points

* human immunodeficiency virus infection or immunosuppressive treatment. ** hemoglobin ≤ 9.2 g/dL, leukocytes ≤ 5000/mm^3^, platelets ≤ 110,000/mm^3^.

**Table 3:** Hemophagocytic lymphohistiocytosis (HLH) probability according to the HScore (adapted from Fardet et al. [39]).

HScore	HLH Probability %
250	>99
240	99
230	98
220	96
210	93
200	88
190	80
180	70
170	54
160	40
150	25
140	16
130	9
120	5
110	3
100	1
90	<1

**Table 4:** The 2016 classification criteria for hemophagocytic lymphohistiocytosis (HLH) (adapted from Ravelli et al. [42]).

Fever
SJIA suspected/proved
Hyperferritinemia > 684 ng/mL
AND ANY 2 OF THE FOLLOWING:
Thrombocytopenia < 181,000/mm^3^
Aspartate aminotransferase > 48 UI/L
Triglycerides > 156 mg/dL
Fibrinogen ≤ 360 mg/dL

SJIA: systemic juvenile idiopathic arthritis.

**Table 5:** Case study 1: Two-month-old male infant with hemophagocytic lymphohistiocytosis. Laboratory results.

	On Admission	Normal Range
Complete blood count		
Leucocytes	3640/μL	5.50–15.50 × 10^3^/μL
Lymphocytes	2290/μL	2–8 × 10^3^/μL
Neutrophils	840/μL	1.5–8.5 10^3^/μL
Platelets	68,000/μL	150,000–450,000/μL
Hemoglobin	5.6 g/dL	11–14 g/dL
Inflammatory markers		
CRP	9.75 mg/L	0–5 mg/L
Procalcitonin	0.256 ng/mL	<0.05 ng/mL
IL-6	16 pg/mL	<7 pg/mL
LDH	402 U/L	120–300 U/L
Ferritin	1342 μg/L	4–67 μg/L
Coagulation		
PT	16.2 s	11.3–15.6 s
APTT	35.2 s	24–37 s
INR	1.24	0.84–1.2
Fibrinogen	77 mg/dL	160–390 mg/dL
D-dimers	1.83 μg/mL	0–0.5 μg/mL
Liver function		
AST	33.2 U/L	2–48 U/L
ALT	15.3 U/L	2–29 U/L
Kidney function		
Creatinine	0.12 mg/dL	<0.47 mg/dL
BUN	12.2 mg/dL	<39 mg/dL
Triglycerides	360 mg/dL	40–150 mg/dL

CRP: C-reactive protein; IL-6: interleukin 6; LDH: lactate dehydrogenase; PT: prothrombin time; APPT: activated partial thromboplastin time; INR: international normalized ratio; AST: aspartate aminotransferase; ALT: alanine transaminase; BUN: blood urea nitrogen.

**Table 6:** Case study 1: Two-month-old infant with hemophagocytic lymphohistiocytosis. Virology and bacterial culture results.

	Patient’s Results	Normal Range
IgG CMV	16.6 U/mL	<0.5 U/mL—negative >1 U/mL—positive
IgM CMV	0.35 U/mL	<0.7 U/mL—negative >1 U/mL—positive
HIV1 + 2 antibody/antigen combo	0.15 U/mL	<0.9 U/mL—negative >1 U/mL—positive
IgG Parvovirus B19	0.2 U/mL	<2 U/mL—negative >3 U/mL—positive
IgM Parvovirus B19	<0.1 U/mL	<20 U/mL—negative >25 U/mL—positive
VCA-IgG EBV	7.93 U/mL	<0.75 U/mL—negative >1 U/mL—positive
VCA-IgM EBV	0.01 U/mL	<0.5 U/mL—negative >1 U/mL—positive
IgG Measles	641.7 U/mL	<200 U/mL—negative >250 U/mL—positive
IgM Measles	<1.9 U/mL	<20 U/mL—negative >25 U/mL—positive
IgM Rubella	0.26 U/mL	<0.8 U/mL—negative >1 U/mL—positive
PCR SARS-CoV-2	Negative	
PCR EBV	Negative	
Adenovirus fecal antigen	Negative	
Rotavirus fecal antigen	Negative	
Blood culture	Negative	
Stool culture	Negative	
Urine culture	Negative	
Pharyngeal exudate	Negative	

IgG: immunoglobulin G; IgM: immunoglobulin M; CMV: cytomegalovirus; HIV: human immunodeficiency virus; VCA: viral capsid antigen; EBV: Ebstein–Barr virus; PCR: polymerase chain reaction; SARS-CoV-2: severe acute respiratory syndrome coronavirus 2.

**Table 7:** Case study 2: 15-month-old female infant with hemophagocytic lymphohistiocytosis. Laboratory results.

	On Admission	4 H after Admission	Normal Range
Complete blood count			
Leucocytes	1820/μL	6890/μL	5000–20,000/μL
Lymphocytes	170/μL	1000/μL	4000–10,500/μL
Neutrophils	1530/μL	5620/μL	1500–8500/μL
Hemoglobin	8.8 g/dL	6.6 g/dL	11.3–14.1/μL
Platelets	20,000/μL	29,000/μL	150,000–450,000/μL
Inflammatory markers			
CRP	247 mg/L		<5 mg/dL
Procalcitonin	10 ng/mL		<0.05 ng/mL
ESR		6 mm/h	<10 mm/h
Ferritin		4596 μg/L	6–67 μg/L
Coagulation			
Fibrinogen		291.6 mg/dL	194–374 mg/dL
APTT	80 s		21.1–28.7 s
PT	16.2 s		8.7–12.2 s
INR	1.55		0.84–1.2
Biochemistry			
ALT		37 U/L	<29 U/L
AST		303 U/L	<59 U/L
Creatinine		1.48 mg/dL	<0.41 mg/dL
Blood urea nitrogen		78 mg/dL	<18 mg/dL
Tryglicerides		162 mg/dL	<159 mg/dL
Creatine-kinase		1314 U/L	<192 U/L
Creatine-kinase MB		188 U/L	<24 U/L
LDH		2765 U/L	225–600 U/L
Sodium	127.5 mmol/L	128.4 mmol/L	135–148 mmol/L
Potassium	3.75 mmol/L	5.59 mmol/L	3.5–4.5 mmol/L
pH	7.32	7.16	7.31–7.41
HCO_3_	16.3 mmol/L	14.7 mmol/L	22–26 mmol/L
Immunogram			
IgA		36.5 mg/dL	20–100 mg/dL
IgG		422.9 mg/dL	453–916 mg/dL
IgM		61.6 mg/dL	19–146 mg/dL

CRP: C-reactive protein; ESR: erythrocyte sedimentation rate; PT: prothrombin time; APPT: activated partial thromboplastin time; INR: international normalized ratio; AST: aspartate aminotransferase; ALT: alanine transaminase; LDH: lactate dehydrogenase; HCO_3_: bicarbonate; Ig: immunoglobulin.

## Data Availability

No new data were created or analyzed in this study. Data sharing is not applicable to this article.
